# Supplementary material for: Perceptions, attitudes, awareness and perspectives towards sustainability practices and climate change among nurses: a systematic review protocol
Source: BMJ Open. 2023 Oct 5;13(10):e071965. doi: 10.1136/bmjopen-2023-071965 (PMC10565322; doi:10.1136/bmjopen-2023-071965)
Supplement: Supplementary data [file bmjopen-2023-071965supp001.pdf]

Supplementary material 1. Full search strategies for perception, attitude, awareness and perspective towards sustainability practices in relation to climate change among nurses.

Database: PubMed

#1 "Nurses"[Mesh] OR "Nursing"[Mesh] OR "nurs*"[tw]

#2 "Climate Change"[Mesh] OR "Greenhouse Effect"[Mesh] OR "Carbon Footprint"[Mesh] OR "climate change"[tw] OR "sustainab*"[tw] OR "sustainable healthcare"[tw] OR "environmentally responsible healthcare"[tw] OR "global warming"[tw] OR "environmental responsib*"[tw] OR net zero healthcare [tw]

#3 "Survey*"[tw] OR " questionnaire*"[tw] OR "interview*"[tw] OR "observ*"[tw] OR "case stud*"[tw] OR "focus group*"[tw]

#4 "Social Perception"[Mesh] "Attitude"[Mesh] "Awareness"[Mesh] OR "Opinion*" OR "perce*"[tw] OR "aware*"[tw] OR "perspect*"[tw] OR "attitude*"[tw] OR "belie*"[tw] OR "know*"[tw] OR "pract*"[tw]

#5 ("Qualitative Research"[Mesh] OR "mixed method*"[tw] OR qualitative[tw] OR quantitative[tw]

#1 AND #2 AND #3 AND #4 AND #5

Filters: English

Database: PsycINFO

#1 “nurs*”

#2 “climate change” OR “sustainab*” OR “sustainable healthcare” OR “environmentally responsible healthcare” OR “global warming” OR “environmental responsib*” OR “net zero healthcare”

#3 “Survey*” OR “questionnaire*” OR “interview*” OR “observ*” OR “case stud*” OR “focus group*”

#4 “Opinion*” OR “perce*” OR “aware*” OR “perspect*” OR “attitude*” OR “belie*” OR “sustainable pract*” OR “know*” OR “pract*”

#5 “mixed method*” OR “qualitative” OR “quantitative”

#1 AND #2 AND #3 AND #4 AND #5

Filters: English

Database: CINAHL ULTIMATE

#1 “nurs*”

#2 “climate change” OR “sustainab*” OR “sustainable healthcare” OR “environmentally responsible healthcare” OR “global warming” OR “environmental responsib*” OR “net zero healthcare”

#3 “Survey*” OR “questionnaire*” OR “interview*” OR “observ*” OR “case stud*” OR “focus group*”

#4 “Opinion*” OR “perce*” OR “aware*” OR “perspect*” OR “attitude*” OR “belie*” OR “sustainable pract*” OR “know*” OR “pract*”

#5 “mixed method*” OR “qualitative” OR “quantitative”

#1 AND #2 AND #3 AND #4 AND #5

Filters: English

DATABASE: SCOPUS

#1 nurs*

#2 climate change*, which was seen in the search as climate AND change*

#1 AND #2

Filters: English

The above in SCOPUS produced relevant papers and papers which were also noted during the scoping review/searches.
