# Supplementary material for: Perceptions, attitudes, awareness and perspectives towards sustainability practices and climate change among nurses: a systematic review protocol
Source: BMJ Open. 2023 Oct 5;13(10):e071965. doi: 10.1136/bmjopen-2023-071965 (PMC10565322; doi:10.1136/bmjopen-2023-071965)
Supplement: Author's manuscript [file bmjopen-2023-071965.draft_revisions.pdf]

BMJ Open

BMJ Open is committed to open peer review. As part of this commitment we make the peer review history of every article we publish publicly available.

When an article is published we post the peer reviewers' comments and the authors' responses online. We also post the versions of the paper that were used during peer review. These are the versions that the peer review comments apply to.

The versions of the paper that follow are the versions that were submitted during the peer review process. They are not the versions of record or the final published versions. They should not be cited or distributed as the published version of this manuscript.

BMJ Open is an open access journal and the full, final, typeset and author-corrected version of record of the manuscript is available on our site with no access controls, subscription charges or pay-per-view fees (http://bmjopen.bmj.com).

If you have any questions on BMJ Open's open peer review process please email info.bmjopen@bmj.com

BMJ Open

PERCEPTION, ATTITUDE, AWARENESS AND PERSPECTIVE TOWARDS SUSTAINABILITY PRACTICES IN RELATION TO CLIMATE CHANGE AMONG NURSES: A SYSTEMATIC REVIEW PROTOCOL

Journal:	*BMJ Open*
Manuscript ID	bmjopen-2023-071965
Article Type:	Protocol
Date Submitted by the Author:	17-Jan-2023
Complete List of Authors:	Akore Yeboah, Ebenezer; Coventry University, centre for healthcare research<br>Adegboye, Amanda ; Coventry University<br>Kneafsey, Rosie ; Coventry University
Keywords:	PUBLIC HEALTH, Health & safety < HEALTH SERVICES ADMINISTRATION & MANAGEMENT, Systematic Review

SCHOLARONE™
Manuscripts

PERCEPTION, ATTITUDE, AWARENESS AND PERSPECTIVE TOWARDS SUSTAINABILITY PRACTICES IN RELATION TO CLIMATE CHANGE AMONG NURSES: A SYSTEMATIC REVIEW PROTOCOL

Ebenezer Akore Yeboah (corresponding author): akoreyeboe@uni.coventry.ac.uk

Centre for Healthcare Research, Coventry University, UK

Amanda Rodrigues Amorim Adegboye: ad6287@coventry.ac.uk

Rosie Kneafsey: aa9398@coventry.ac.uk

ABSTRACT

Introduction: Climate change has been described as the single biggest menace humanity is dealing with in this century. It is widely accepted that contemporary human activities are the major causes of climate change. The impact of climate change on human health is significant and as such is a critical issue. Healthcare professionals have the potential to promote climate advocacy and mitigation through a collective effort or individual actions. However, research suggests that nurses may not be aware of the potential to effect positive action. Hence, this review aims to synthesise evidence concerning perception, attitude, awareness, and perspectives of sustainable nursing practice in relation to climate change among nurses.

Methods and Analysis: The JBI methodology for mixed methods systematic review will be used for this proposed systematic review. And will be reported in accordance with Preferred Reporting Items for Systematic Reviews and Meta Analyses (PRISMA) guidelines. CINAHL, PsycINFO, SCOPUS, and PUBMED databases will be searched. Data appraisal to be done with JBI and MMAT critical appraisal tool. Data synthesis and integration will follow JBI convergent integrated approach.

Ethics and Dissemination: Ethical approval would be sought from the Ethics Committee, Coventry University, UK. Dissemination of findings will be done via journals, conferences and seminars.

For peer review only

Strength and limitations

- This study will be limited to papers published in English.
- Hence the risk of missing out relevant information in other languages.
- However, rigour and transparency will be observed in the review process.

INTRODUCTION

Climate change has been described as the single biggest menace humanity is dealing with in this century [1]. Climate change is defined as 'the systematic change in the long term state of the atmosphere over multiple decades or longer'[2]. In the 19th century, natural events such as changes in incoming solar radiation, volcanoes and changes in global biogeochemical cycles were identified by scientists as the major causes of climate change [3]. However, contemporary human activities such as combustion of fossil fuel for energy production and use, deforestations, atmospheric aerosols, change in land use and emission of other gases such as water vapour are the major causes of climate change, established through scientific consensus [3].

The impact of climate change on human health is significant and as such is a critical issue [4]. It is widely accepted that those who suffer most are those who contribute little to greenhouse gas emissions [5]. They are mostly the vulnerable ones due to geographic placement, social isolation, and economic disadvantage. Climate change is estimated to cause an excess death of about 250,000 additional between 2030 and 2050, from heat exhaustion in elderly people, malnutrition among children, malaria and diarrhoea [4].

Globally, health professionals are acknowledging and responding to the damage caused by climate change in relation to health [6]. Healthcare systems account for 4.4% of the global net CO2 emission. It has been identified that if the healthcare sector were a country, it would rank the fifth-largest producer of greenhouse gases on the planet [7]. This statement indicates the significant climate impact of health care. It contributes through emissions and use of carbon compounds for the good of human health. For example, the UK, National Health Service (NHS), account for 5.4% of the UK greenhouse emissions and this is higher than the global average of the healthcare system [7].

For peer review only

Healthcare professionals have the potential to promote climate advocacy and mitigation through a collective effort or individual actions[8]. However, research suggests that nurses may not be aware of the potential to effect positive action [9]. A study in Sweden brought out that nurses exhibited understanding of environmental issues in their local setting, however, comprehensiveness of the global perspective of climate change and environmental issues were inadequate [10]. However, the author's preconceptions of the subjects and limited geographic rich of sample (restricted to one county in Sweden) may limit the generalisability of its findings [10]. Also, a study in China showed that 76% of nurses knew that climate change would affect public health. However, more than 50% did not know their nursing practice could be related to emission of carbon compounds and subsequently climate change [11].

Another study including American nurses found that they believe climate change is happening and that they have a responsibility as health workers to address the health effects of climate change. But these nurses did not have the aptness to these climate change health implication [9].

As nurses form the largest part of the health professional group, it is urging for nurses to find mitigating factors to climate impact in practice as well as infuse sustainability in the nursing curricula [12]. For this, a critical and systematic evolution of the literature on climate change and nursing is required. It is important to understanding nurses' perspective, attitudes and behaviours which can inform nursing curriculum development and professional practice on climate change.

There is an ongoing integrative review protocol on awareness and attitude toward sustainability and climate change among students and educators in nursing [13] however, there is no systematic review focusing on the perspectives of professional nurses. Studies have used different samples, designs and reached different conclusions, therefore it seems relevant to systematically synthesis the literature and critical appraise the studies in this field. Therefore, this review aim to synthesise evidence concerning perception, attitude, awareness, and perspectives of sustainable nursing practice in relation to climate change among nurses.

METHOD

For peer review only

DESIGN

A mixed method systematic review will be conducted to answer the research question related to the phenomena of interest of this study. Mixed method systematic review merges and homogenise findings of quantitative and qualitative studies within a single review [14]. Mixed method review is useful in producing systematic review of direct importance or significance to policy makers and practitioners [15]. This design is appropriate and suitable when the researcher wants to broaden the conceptualisation of evidence that is methodologically inclusive. It synthesizes evidence that will be available to wider range of consumers hence it will enable comprehensiveness around climate change and sustainability [16]. The updated JBI methodology for mixed methods systematic review [17] will be used for this proposed systematic review. This review will be reported in accordance with Preferred Reporting Items for Systematic Reviews and Meta Analyses (PRISMA) guidelines [18]. The review protocol is registered in the Open Science Framework (OSF) and registration DOI 10.17605/OSF.IO/8H3TC.

Identification of problem

The SPIDER (Sample, Phenomenon of Interest, Design, Evaluation and Research type) framework will be used to formulate the research question and determine the criteria for inclusion and exclusion. Table 1.

Table 1. Inclusion and exclusion criteria in the literature search

Domains	Inclusion	Exclusion
Sample	▪ Studies/ publication /articles focused on registered or qualified nurses regardless of work setting<br>▪ Can include other health professionals as long as findings reported nurses separately.	▪ Studies/ publication /articles on climate change focusing on nursing students and/or nursing educators.<br>▪ Studies on other healthcare professional e.g. care assistant, doctor etc.
Phenomenon of interest	▪ Climate change, environmental sustainability, net zero healthcare and nursing practice or any related terms to these	▪ Studies that do not include climate change or sustainable environmental practice or any related term

For peer review only - http://bmjopen.bmj.com/site/about/guidelines.xhtml

Design	▪ Empirical research studies using any research design e.g., interview, survey, focus group etc.<br>▪ Published literature, governmental reports, technical documents, manuals, conference abstracts, non-governmental reports on net zero healthcare (or climate change) with relation to nursing (which are primary or empirical study).	▪ Commentaries, press releases, speeches, letters to the editor are excluded<br>▪ All publication on the phenomena of interest which are not empirical research.
Evaluation	▪ Studies exploring the perception, OR attitude, OR awareness (or knowledge) OR the perspective on climate change and related terms	
Research type	❖ Qualitative, quantitative or mixed methods approach of approach of primary research	❖ Secondary research

All studies regardless of the date of publication will be included. Studies across the globe or any country will be included but limited to publications in English.

Searching Literature

Search strategy

A preliminary scoping search was done to identify articles on the study topic. A full search strategy was developed after going through various titles and abstract of relevant articles and their index terms. Table 2.

Information source

The database to be searched include; CINAHL, PsycINFO, SCOPUS and PUBMED. These data bases have been selected due to their broad range of literature in the fields of health and social sciences, nursing and climate change.

Table 2: Search terms

	Concept	Truncated terms

For peer review only

Sample	"nursing" OR "nurses"	"nurs*"
Phenomenon of interest	"climate change" OR "sustainability" OR "sustainable healthcare" OR "environmentally responsible healthcare" OR "global warming" OR "environmental responsibility" OR "net zero healthcare"	"climate change" OR "sustainab*" OR "sustainable healthcare" OR "environmentally responsible healthcare" OR "global warming" OR "environmental responsib*" OR "net zero healthcare"
Design	"Survey" OR " questionnaire" OR "interviews" OR "observational study" OR "case study" OR "focus group discussion"	"Survey*" OR " questionnaire*" OR "interview*" OR "observ*" OR "case stud*" OR "focus group*"
Evaluation	"perception" OR "awareness" OR "perspectives" OR "attitude" OR "beliefs" OR "knowledge" OR "opinion" OR "sustainable practice" OR "practice"	"Opinion*" OR "perce*" OR "aware*" OR "perspect*" OR "attitude*" OR "belie*" OR "sustainable pract*" OR "know*" OR "pract*"
Research type	"mixed methods" OR "qualitative" OR "quantitative"	"mixed method*" OR "qualitative" OR "quantitative"

Study selection

The search results from each database will be imported in EndNote reference manager to remove duplicates and then transferred to Rayyan web tool where screening based on the inclusion and exclusion criteria will take place. In the initial phase, 20% of the studies will be screened by the three reviewers (EAY, AA, RK) independently based on title and abstract. This independent screening process will reduce the risk of bias and ensure consistency in the review process [19]. Once the initial screening phase is done, two reviewers (EAY, AA) will continue the title and abstract screening on the remaining studies. Any studies selected at this stage will then be subjected to full text screening by the same two reviewers (EAY, AA) [13]. Any differences between the two reviewers will be resolved through discussion or by the third reviewer (RK).

For peer review only

During this second search, bibliography or reference lists of the studies which were included will be manually reviewed. Any reason for excluding a paper during the full text assessment that do not meet the inclusion criteria will be recorded and reported in the systematic review findings. In instances of disagreement between the two reviewers in the second phase of screening the full text, consensus will be reached through discussion or third reviewer. All members of the review team (EAY, AA, RK) will inspect and assess the final list of papers that met the inclusion criteria before the progressing to nest stages of the review methodology. A PRISMA flow diagram will be designed to show the completed search [20].

Assessment of methodological quality.

The standardised JBI qualitative critical appraisal tool will be used to assess qualitative papers that will meet inclusion criteria [21]. The quantitative and mixed method papers will be methodologically appraised with the Mixed Methods Assessment Tool (MMAT) version 2018 [22]. These methodological appraisal will be done by the two review members (EAY, AA) independently prior to data extraction.

The quality appraisal of the included papers will be compared and discussed to determine the overall quality score and in situations of dissimilarity, it would be discussed collaboratively among the review team, and to employ triangulation or independent reviewer as a measure of credibility or trustworthiness [19]. These will be done to reduce any risks of bias and provide compelling evidence for the study. Regardless of the methodological quality, all included studies will undergo data extraction and synthesis (where possible). The results of the appraisal will summarised in a table and reported in the review.

Data extraction

Both the qualitative and quantitative data will be extracted from the included papers by two reviewers (EAY, AA) using the standardised JBI data extraction tools [21]. Any discrepancies will be resolved discussion among the two reviewers or with triangulation. Authors of papers will be contacted for missing or additional data, if required [21].

Data transformation

For the purpose of integration, the extracted data will need to be transformed into mutually compatible forms [23]. In this review, the retrieved quantitative data will undergo qualitizing

For peer review only

thus extracted quantitative data will be converted into qualitative data. This will involve conversion of quantitative data into textual description thus themes, categories etc, in order to be able to answer the review question. Qualitizing is chosen to reduce the risk of error as opposed to quantizing [24].

Data synthesis and integration

The JBI convergent integrated approach will be adopted to synthesize the transformed data [21]. This involves combination of extracted data from quantitative and qualitative or mixed method studies. The qualitized data will be assembled with qualitative and then categorised. The categorised data will be pooled together based on similarity in meaning to produce a set of integrated finding [17].

Patient and Public Involvement

None

Discussion

This mixed method review will seek to enrich the conceptualisation of evidence that methodologically inclusive and synthesize data that will be available to the nursing community and policy makers. This will help synthesize the existing body of literature related to the perception, attitude, awareness and perspective towards sustainability practices in relation to climate among nurses. The finding of the review could be significant to hospital managers, nurse leaders etc. when planning projects on climate sustainability or greening hospital as nurses form the largest part of the health professional group [12]. It is significant to translate evidence from research into practice so that professionals are well informed of current information to encourage incorporation of sustainability in relation to climate change in the nursing practice [25]. The review will be disseminated in journals, conferences, and with relevant institutions to improve upon practice and sharing of knowledge for the benefit of the global population. Climate change and sustainability are becoming ubiquitous in all spheres of life as the world is experiencing the impact of this phenomenon; ranging from heatwaves, flood, and drought to climate disease. There is an establishment of incongruence in the issue of climate change and practice among nurses [26] hence doing a mixed method systematic review will bring out more comprehensiveness to the activities of nurses and address their role in mitigating this menace on humanity.

For peer review only

Conclusion

The findings of this mixed method review might identify the potential gaps in the practice of sustainability in relation to climate change among nurses and will inform policy makers thus leaders of hospital, management on the need for a relook into policies and the significance of professional development of nurses. In addition, it might increase the comprehensiveness around climate change and environmentally responsible healthcare practice among nurses for the good of humanity and the health of the planet.

Ethics

Ethical approval would be sought from the Ethics Committee, Coventry University, UK.

Acknowledgement

Thank you to the Centre for Healthcare Research, Coventry University, for funding the PhD study. No fund/grant number.

Funding

No source of external funding.

Conflict of interest

The authors declared no potential conflicts of interest.

Author Contributions

Ebenezer Akore Yeboah, Amanda Rodrigues Amorim Adegboye, and Rosie Kneafsey, all contributed to the conception, design, drafting manuscript, revising and approval of the final version to be published. However, Ebenezer Akore Yeboah is accountable for all aspect of the work.

Reference

1. Watts, N., et al., *The 2020 report of the Lancet Countdown on health and climate change: responding to converging crises.* 2021. **397**(10269): p. 129-170.

For peer review only

2. Knowlton, K., C. Sorensen, and J. Lemery, *Global climate change and human health: from science to practice*. 2021: John Wiley & Sons.
3. Geneva, S.J.W.G.I.C.t.t.I.F.A.R.C.C., *Intergovernmental Panel on Climate Change, 2014.* 2013. **8**.
4. WHO, *climate change.* 2022.
5. Lemery, J., et al., *The great procrastination*. 2014, HeinOnline. p. 1.
6. WHO, *climate change and health.* World Health Organisation, 2021.
7. Harm, H.C.W., *Health Care's Climate Footprint—How the Health Sector Contributes to the Global Climate Crisis and Opportunities for Action*. 2019, ARUP London, UK.
8. Whitmee, S., et al., *Safeguarding human health in the Anthropocene epoch: report of The Rockefeller Foundation–Lancet Commission on planetary health.* 2015. **386**(10007): p. 1973-2028.
9. Polivka, B.J., R.V. Chaudry, and J. Mac Crawford, *Public Health Nurses' Knowledge and Attitudes Regarding Climate Change.* Environmental Health Perspectives, 2012. **120**(3): p. 321-325.
10. Anåker, A., et al., *Nurses' perceptions of climate and environmental issues: a qualitative study.* Journal of Advanced Nursing (John Wiley & Sons, Inc.), 2015. **71**(8): p. 1883-1891.
11. Xiao, J., et al., *Nurses' knowledge and attitudes regarding potential impacts of climate change on public health in central of China.* International Journal of Nursing Sciences, 2016. **3**(2): p. 158-161.
12. Lilienfeld, E., et al., *Addressing climate change through a nursing lens within the framework of the United Nations Sustainable Development Goals.* Nursing Outlook, 2018. **66**(5): p. 482-494.
13. Aronsson, J., et al., *Awareness and attitudes towards sustainability and climate change amongst students and educators in nursing: A systematic integrative review protocol.* Nursing Open, 2022. **9**(1): p. 839-844.
14. Harden, A.J.F., *Mixed-methods systematic reviews: Integrating quantitative and qualitative findings.* 2010. **25**: p. 1-8.
15. Pearson, A., et al., *A mixed-methods approach to systematic reviews.* 2015. **13**(3): p. 121-131.
16. Sandelowski, M., et al., *Text-in-context: a method for extracting findings in mixed-methods mixed research synthesis studies.* 2013. **69**(6): p. 1428-1437.
17. Stern, C., et al., *Methodological guidance for the conduct of mixed methods systematic reviews.* JBI evidence implementation, 2021. **19**(2): p. 120-129.
18. Page, M.J., et al., *The PRISMA 2020 statement: an updated guideline for reporting systematic reviews.* 2021. **10**(1): p. 1-11.
19. Polit, D. and C. Beck, *Essentials of nursing research: Appraising evidence for nursing practice*. 2020: Lippincott Williams & Wilkins.
20. Moher, D.J.P.r.i.f.s.r. and M.T.P.s. bMJ, *liberati a, Tetzlaff J, altman Dg; The PrisMa group.* 2009. **339**: p. b2535.
21. Munn, Z., et al., *The development of software to support multiple systematic review types: the Joanna Briggs Institute System for the Unified Management, Assessment and Review of Information (JBI SUMARI).* 2019. **17**(1): p. 36-43.
22. Hong, Q.N., et al., *The Mixed Methods Appraisal Tool (MMAT) version 2018 for information professionals and researchers.* 2018. **34**(4): p. 285-291.
23. Voils, C., et al., *A Bayesian method for the synthesis of evidence from qualitative and quantitative reports: the example of antiretroviral medication adherence.* 2009. **14**(4): p. 226-233.
24. Institute, J.B.I.J.A.T.J.B., *The Joanna Briggs Institute Reviewers' manual 2014: methodology for JBI mixed methods systematic reviews.* 2014.

For peer review only

25. Munn, Z., et al., *Evidence transfer: ensuring end users are aware of, have access to, and understand the evidence.* 2018. **16**(2): p. 83-89.

26. Kalogirou, M.R., et al., *Nurses' perspectives on climate change, health and nursing practice.* Journal of Clinical Nursing (John Wiley & Sons, Inc.), 2020. **29**(23/24): p. 4759-4768.

For peer review only

PRISMA-P 2015 Checklist

This checklist has been adapted for use with protocol submissions to *Systematic Reviews* from Table 3 in Moher D et al: Preferred reporting items for systematic review and meta-analysis protocols (PRISMA-P) 2015 statement. *Systematic Reviews* 2015 **4**:1

Section/topic	#	Checklist item	Information reported: Yes	Information reported: No	Line number(s)
**ADMINISTRATIVE INFORMATION**					
**Title**					
Identification	1a	Identify the report as a protocol of a systematic review	☒	☐	
Update	1b	If the protocol is for an update of a previous systematic review, identify as such	☐	☒	
**Registration**	2	If registered, provide the name of the registry (e.g., PROSPERO) and registration number in the Abstract	☒	☐	
**Authors**					
Contact	3a	Provide name, institutional affiliation, and e-mail address of all protocol authors; provide physical mailing address of corresponding author	☒	☐	
Contributions	3b	Describe contributions of protocol authors and identify the guarantor of the review	☒	☐	
**Amendments**	4	If the protocol represents an amendment of a previously completed or published protocol, identify as such and list changes; otherwise, state plan for documenting important protocol amendments	☐	☒	
**Support**					
Sources	5a	Indicate sources of financial or other support for the review	☒	☐	
Sponsor	5b	Provide name for the review funder and/or sponsor	☒	☐	
Role of sponsor/funder	5c	Describe roles of funder(s), sponsor(s), and/or institution(s), if any, in developing the protocol	☒	☐	
**INTRODUCTION**					
**Rationale**	6	Describe the rationale for the review in the context of what is already known	☒	☐	
**Objectives**	7	Provide an explicit statement of the question(s) the review will address with reference to participants, interventions, comparators, and outcomes (PICO)	☒	☐	
**METHODS**					

BioMed Central
The Open Access Publisher

Section/topic	#	Checklist item	Information reported Yes	No	Line number(s)
**Eligibility criteria**	8	Specify the study characteristics (e.g., PICO, study design, setting, time frame) and report characteristics (e.g., years considered, language, publication status) to be used as criteria for eligibility for the review	☒	☐	
**Information sources**	9	Describe all intended information sources (e.g., electronic databases, contact with study authors, trial registers, or other grey literature sources) with planned dates of coverage	☒	☐	
**Search strategy**	10	Present draft of search strategy to be used for at least one electronic database, including planned limits, such that it could be repeated	☒	☐	
STUDY RECORDS					
Data management	11a	Describe the mechanism(s) that will be used to manage records and data throughout the review	☒	☐	
Selection process	11b	State the process that will be used for selecting studies (e.g., two independent reviewers) through each phase of the review (i.e., screening, eligibility, and inclusion in meta-analysis)	☒	☐	
Data collection process	11c	Describe planned method of extracting data from reports (e.g., piloting forms, done independently, in duplicate), any processes for obtaining and confirming data from investigators	☒	☐	
**Data items**	12	List and define all variables for which data will be sought (e.g., PICO items, funding sources), any pre-planned data assumptions and simplifications	☒	☐	
**Outcomes and prioritization**	13	List and define all outcomes for which data will be sought, including prioritization of main and additional outcomes, with rationale	☒	☐	
**Risk of bias in individual studies**	14	Describe anticipated methods for assessing risk of bias of individual studies, including whether this will be done at the outcome or study level, or both; state how this information will be used in data synthesis	☒	☐	
DATA					
**Synthesis**	15a	Describe criteria under which study data will be quantitatively synthesized	☐	☒	
	15b	If data are appropriate for quantitative synthesis, describe planned summary measures, methods of handling data, and methods of combining data from studies, including any planned exploration of consistency (e.g., I^2, Kendall's tau)	☐	☒	
	15c	Describe any proposed additional analyses (e.g., sensitivity or subgroup analyses, meta-regression)	☐	☒	
	15d	If quantitative synthesis is not appropriate, describe the type of summary planned	☒	☐	
**Meta-bias(es)**	16	Specify any planned assessment of meta-bias(es) (e.g., publication bias across studies, selective reporting within studies)	☒	☐	
**Confidence in cumulative evidence**	17	Describe how the strength of the body of evidence will be assessed (e.g., GRADE)	☒	☐	

BioMed Central The Open Access Publisher

BMJ Open

PERCEPTIONS, ATTITUDES, AWARENESS AND PERSPECTIVES TOWARDS SUSTAINABILITY PRACTICES AND CLIMATE CHANGE AMONG NURSES: A SYSTEMATIC REVIEW PROTOCOL

Journal:	*BMJ Open*
Manuscript ID	bmjopen-2023-071965.R1
Article Type:	Protocol
Date Submitted by the Author:	08-May-2023
Complete List of Authors:	Akore Yeboah, Ebenezer; Coventry University, Research Centre for Healthcare and Communities<br>Adegboye, Amanda ; Coventry University, Centre for Agroecology, Water and Resilience<br>Kneafsey, Rosie ; Coventry University, Research Centre for Healthcare and Communities
**Primary Subject Heading**:	Nursing
Secondary Subject Heading:	Nursing
Keywords:	PUBLIC HEALTH, Health & safety < HEALTH SERVICES ADMINISTRATION & MANAGEMENT, Systematic Review

SCHOLARONE™
Manuscripts

PERCEPTIONS, ATTITUDES, AWARENESS AND PERSPECTIVES TOWARDS SUSTAINABILITY PRACTICES AND CLIMATE CHANGE AMONG NURSES: A SYSTEMATIC REVIEW PROTOCOL

Ebenezer Akore Yeboah[1] (corresponding author): ebenezer.akore@gmail.com

Amanda Rodrigues Amorim Adegboye[2]: ad6287@coventry.ac.uk

Rosie Kneafsey[1]: aa9398@coventry.ac.uk

1. Research Centre for Healthcare and Communities, Coventry University, UK
2. Centre for Agroecology, Water and Resilience, Coventry University, UK

For peer review only

ABSTRACT

Introduction: Climate change has been described as the most significant threat to humanity and human health to have emerged this century. It is widely accepted that contemporary human activities are the major causes of climate change. It is also acknowledged that damaging human activities could be amenable to change through pro-active environmental behaviours. Healthcare professionals have the potential to promote climate advocacy and mitigation through collective effort and individual actions. However, research suggests that nurses may not be aware of their potential to effect positive action. This review will synthesise evidence regarding nurses' perceptions, attitudes, awareness, and perspectives towards sustainable nursing practices and climate change.

Methods and Analysis: The JBI methodology for mixed methods systematic reviews will be applied to this proposed systematic review. It will be reported in accordance with the Preferred Reporting Items for Systematic Reviews and Meta Analyses (PRISMA) guidelines. CINAHL, PsycINFO, SCOPUS, and PUBMED databases will be searched. Data appraisal will be completed using the JBI and MMAT critical appraisal tool. Data synthesis and integration will follow the JBI convergent integrated approach.

Ethics and Dissemination: In compliance with University ethics requirements for secondary research and post-graduate researchers, ethical approval will be sought from the Coventry University Ethics Committee, UK. Dissemination of findings will be achieved through peer-review publications, conference presentations and seminars with local, national and international audiences.

For peer review only - http://bmjopen.bmj.com/site/about/guidelines.xhtml

Strength and limitations

- ❖ This study will be limited to papers published in English.
- ❖ There is a risk that relevant information in other languages could be missed.
- ❖ Rigour and transparency will be observed in the review process.

For peer review only

INTRODUCTION

Climate change has been described as the most significant threat this century to humanity and the greatest source of harm to human health [1]. Climate change is defined as 'the systematic change in the long term state of the atmosphere over multiple decades or longer'[2]. In the 19th century, natural events such as changes in incoming solar radiation, volcanoes and changes in global biogeochemical cycles were identified by scientists as the major causes of climate change [3]. However, scientific consensus has established that contemporary human activities such as combustion of fossil fuel for energy production and energy use, deforestations, atmospheric aerosols, change in land use and emission of other gases such as water vapour and other greenhouse gases are the major causes of climate change[3].

The impact of climate change on human health is significant and as such is a critical issue [4]. It is widely accepted that the populations which suffer most are those which contribute little to greenhouse gas emissions [5]. Such populations are particularly vulnerable due to geographic placement, social isolation, and economic disadvantage. The documented impact of heatwaves, floods, drought and climate change related disease have been devastating. For example, it is estimated that climate change will cause an additional 250,000 deaths across the globe between 2030 and 2050, from heat exhaustion in elderly people, malnutrition among children, malaria and diarrhoea [4].

Globally, health professionals are acknowledging and responding to the damage caused by climate change and the impact on health [6]. At the same time, healthcare systems themselves account for 4.4% of the global net CO2 emissions thus rendering significant climate impact, both through carbon emissions and use of carbon compounds for human health benefit. Indeed,

For peer review only - http://bmjopen.bmj.com/site/about/guidelines.xhtml

it has been identified that if the healthcare sector were a country, it would rank the fifth-largest producer of greenhouse gases on the planet [7]. More specifically for the UK, the National Health Service (NHS) accounts for 5.4% of the UK greenhouse emissions and this is higher than the global average of the healthcare system [7].

Healthcare professionals have the potential to promote climate advocacy and mitigation through collective effort and individual actions[8]. However, research suggests that nurses may not be aware of the potential to effect positive action [9]. A study in Sweden identified that whilst nurses exhibited an understanding of environmental issues in their local setting, a comprehensive global perspective on climate change and environmental issues was less developed [10]. In contrast, a study in China showed that whilst 76% of nurses knew that climate change would affect public health, more than 50% did not know that their own local nursing practice could be related to carbon compound emissions and subsequently climate change [11]. Across continents, a study including American nurses found that they clearly believed the present threat of climate change and recognised their responsibility as health workers to address the health effects of climate change [9].

It is known that nurses form the largest proportion of the healthcare workforce and are a significant professional force. As such, practicing environmentally responsible healthcare as well as embedding the concepts of sustainability and climate actions into nursing curricula could be important in the fight against climate change [12]. There exists an ongoing integrative review protocol exploring awareness and attitudes of student nurses and educators toward sustainability and climate change [13]. However, there is no systematic review focusing on the perspectives of registered/ qualified nurses. Studies have used different samples, research designs and have reached different conclusions. Therefore, this review aims to systematically synthesise evidence concerning nurses' perceptions, attitudes, awareness, and perspectives of sustainable nursing practice and climate change.

METHOD

For peer review only - http://bmjopen.bmj.com/site/about/guidelines.xhtml

DESIGN

A mixed method systematic review will be conducted to answer the research question related to the phenomena of interest in this study. Mixed method systematic review merges and homogenise findings of quantitative and qualitative studies within a single review [14]. Mixed methods reviews are useful in producing systematic reviews of direct importance or significance to policy makers and practitioners [15]. This design is also appropriate and suitable when the researcher wants to broaden the conceptualisation of evidence that is methodologically inclusive. It synthesizes evidence that will be available to a wider range of consumers. Hence, this approach enables a comprehensiveness exploration of the literature related to nursing, climate change and sustainability [16]. The updated JBI methodology for mixed methods systematic reviews [17] will be used for this systematic review. It will be reported in accordance with the Preferred Reporting Items for Systematic Reviews and Meta Analyses (PRISMA) guidelines [18]. The review protocol is registered in the Open Science Framework (OSF) and registration DOI 10.17605/OSF.IO/8H3TC.

Identification of problem

The SPIDER (Sample, Phenomenon of Interest, Design, Evaluation and Research type) framework has been used to formulate the research question and determine the criteria for inclusion and exclusion. Table 1.

Table 1. Inclusion and exclusion criteria in the literature search

Domains	Inclusion	Exclusion
Sample	▪ Studies/ publication /articles focused on registered or qualified nurses regardless of work setting<br>▪ Can include other health professionals as long as findings reported nurses separately.	▪ Studies/ publication /articles on climate change focusing on nursing students and/or nursing educators.<br>▪ Studies on other healthcare professional e.g. care assistant, doctor etc.
Phenomenon of interest	▪ Climate change, environmental sustainability, net zero healthcare and nursing practice or any related terms to these	▪ Studies that do not include climate change or sustainable environmental practice or any related term

For peer review only - http://bmjopen.bmj.com/site/about/guidelines.xhtml

Design	▪ Empirical research studies using any research design e.g., interview, survey, focus group etc.<br>▪ Published literature, governmental reports, technical documents, conference abstracts, manuals, on net zero healthcare (or climate change) with relation to nursing (which are primary or empirical study).	▪ Commentaries, press releases, speeches, letters to the editor are excluded<br>▪ All publications relating to the phenomena of interest which are not empirical research.
Evaluation	▪ Studies exploring the perception, OR attitude, OR awareness (or knowledge) OR the perspective on climate change and related terms	
Research type	❖ Qualitative, quantitative or mixed methods approaches to primary research	❖ Secondary research

All studies regardless of the date of publication will be included. Studies across the globe or any country will be included but limited to publications in English.

Searching Literature

Search strategy

A preliminary scoping search was done to identify articles on the study topic. A full search strategy was developed after filtering through various titles and abstract of relevant articles and their index terms. Table 2.

Information source

The databases to be searched include; CINAHL, PsycINFO, SCOPUS and PUBMED. These data bases have been selected due to their broad range of literature in the fields of health and social sciences, nursing and climate change.

Table 2: Search terms

	Concept	Truncated terms
Sample	"nursing" OR "nurses"	"nurs*"

For peer review only - http://bmjopen.bmj.com/site/about/guidelines.xhtml

Phenomenon of interest	"climate change" OR "sustainability" OR "sustainable healthcare" OR "environmentally responsible healthcare" OR "global warming" OR "environmental responsibility" OR "net zero healthcare"	"climate change" OR "sustainab*" OR "sustainable healthcare" OR "environmentally responsible healthcare" OR "global warming" OR "environmental responsib*" OR "net zero healthcare"
Design	"Survey" OR " questionnaire" OR "interviews" OR "observational study" OR "case study" OR "focus group discussion"	"Survey*" OR " questionnaire*" OR "interview*" OR "observ*" OR "case stud*" OR "focus group*"
Evaluation	"perception" OR "awareness" OR "perspectives" OR "attitude" OR "beliefs" OR "knowledge" OR "opinion" OR "sustainable practice" OR "practice"	"Opinion*" OR "perce*" OR "aware*" OR "perspect*" OR "attitude*" OR "belie*" OR "sustainable pract*" OR "know*" OR "pract*"
Research type	"mixed methods" OR "qualitative" OR "quantitative"	"mixed method*" OR "qualitative" OR "quantitative"

Study selection

The search results from each database will be imported in EndNote reference manager to remove duplicates and then transferred to Rayyan web tool where screening based on the inclusion and exclusion criteria will take place. In the initial phase, 20% of the studies will be screened by the three reviewers (EAY, AA, RK) independently based on title and abstract. This independent screening process will reduce the risk of bias and ensure consistency in the review process [19]. Once the initial screening phase is done, two reviewers (EAY, AA) will continue the title and abstract screening on the remaining studies. Any studies selected at this stage will then be subjected to full text screening by the same two reviewers (EAY, AA) [13]. Any differences between the two reviewers will be resolved through discussion or by the third reviewer (RK).

For peer review only - http://bmjopen.bmj.com/site/about/guidelines.xhtml

During this second search, bibliography or reference lists of the studies which were included will be manually reviewed. Any reason for excluding a paper during the full text assessment that does not meet the inclusion criteria will be recorded and reported in the systematic review findings. In instances of disagreement between the two reviewers in the second phase of screening the full text, consensus will be reached through discussion or third reviewer. All members of the review team (EAY, AA, RK) will inspect and assess the final list of papers that met the inclusion criteria before the progressing to the next stages of the review methodology. A PRISMA flow diagram will be designed to show the completed search [20].

For peer review only

Assessment of methodological quality.

The standardised JBI qualitative critical appraisal tool will be used to assess qualitative papers that meet the inclusion criteria [21]. The quantitative and mixed method papers will be methodologically appraised with the Mixed Methods Assessment Tool (MMAT) version 2018 [22]. These methodological appraisals will be done by the two review members (EAY, AA) independently, prior to data extraction.

The quality appraisal of the included papers will be compared and discussed to determine the overall quality score and in situations of dissimilarity, it would be discussed collaboratively among the review team, employing triangulation or an independent reviewer as a measure of credibility or trustworthiness [19]. This will be done to reduce any risks of bias and provide compelling evidence for the study. Regardless of the methodological quality, all included studies will undergo data extraction and synthesis (where possible). The results of the appraisal will summarised in a table and reported in the review.

Data extraction

Both the qualitative and quantitative data will be extracted from the included papers by two reviewers (EAY, AA) using the standardised JBI data extraction tools [21]. Any discrepancies will be resolved through discussion among the two reviewers or with triangulation. Authors of papers will be contacted for missing or additional data, if required [21].

Data transformation

For the purpose of integration, the extracted data will need to be transformed into mutually compatible forms [23]. In this review, the retrieved quantitative data will undergo qualitizing

For peer review only - http://bmjopen.bmj.com/site/about/guidelines.xhtml

[24]. In this way, extracted quantitative data will be converted into qualitative data. This will involve conversion of quantitative data into textual description thus themes, categories etc, in order to be able to answer the review question. Qualitizing is chosen to reduce the risk of error as opposed to quantitizing [24].

Data synthesis and integration

The JBI convergent integrated approach will be adopted to synthesize the transformed data [21]. This involves combining the extracted data from quantitative and qualitative or mixed method studies. The qualitized data will be assembled with qualitative information and then categorised. The categorised data will be pooled together based on similarity in meaning to produce a set of integrated finding [17].

Patient and Public Involvement

None

Discussion

This mixed method review will enrich the conceptualisation of evidence so that methodologically inclusive and synthesized data will be available to the nursing community and policy makers. This review will synthesize the existing body of literature related to nurses' perceptions, attitudes, awareness and perspectives towards sustainability practices in relation to climate change. The findings could be significant to hospital managers, nurse leaders and green or sustainability champions when planning environmentally responsible healthcare projects. For example, relating to sustainable healthcare practices, meeting net-zero goals or greening hospitals. Since nurses form the largest part of the healthcare workforce and present a significant professional force, translating evidence from research into practice and promoting nurse engagement in this aspect of healthcare could be impactful [12, 25]. The review will be disseminated in journals, conferences, and with relevant institutions to improve practice and share knowledge for the benefit of the global population. Climate change and sustainability are becoming ubiquitous in all spheres of life as the world experiences the impact of this phenomenon.

Conclusion

For peer review only - http://bmjopen.bmj.com/site/about/guidelines.xhtml

The findings of this mixed method review will be to raise awareness of sustainable healthcare actions and identify opportunities to accelerate the implementation of environmentally responsible nursing practices. The review will also promote dialogue with policy makers, nursing and healthcare leaders regarding the benefit of further mobilising nurse engagement in climate actions and policies.

Ethics

Ethical approval will be sought from the University Ethics Committee, Coventry University, UK.

Acknowledgement

Thank you to all the staff of Research Centre for Healthcare and Communities, Coventry University, for supporting the PhD study.

Funding

Thank you to the Research Centre for Healthcare and Communities, Coventry University, for funding the PhD study. No fund/grant number.

Conflict of interest

The authors declared no potential conflicts of interest.

Author Contributions

Ebenezer Akore Yeboah, Amanda Rodrigues Amorim Adegboye, and Rosie Kneafsey, all contributed to the conception, design, drafting manuscript, revising and approval of the final version to be published. However, Ebenezer Akore Yeboah is accountable for all aspect of the work.

Reference

1. Watts, N., et al., *The 2020 report of the Lancet Countdown on health and climate change: responding to converging crises.* 2021. **397**(10269): p. 129-170.
2. Knowlton, K., C. Sorensen, and J. Lemery, *Global climate change and human health: from science to practice*. 2021: John Wiley & Sons.
3. Geneva, S.J.W.G.I.C.t.t.I.F.A.R.C.C., *Intergovernmental Panel on Climate Change, 2014.* 2013. **8**.

For peer review only - http://bmjopen.bmj.com/site/about/guidelines.xhtml

4. WHO, *climate change.* 2022.
5. Lemery, J., et al., *The great procrastination*. 2014, HeinOnline. p. 1.
6. WHO, *climate change and health.* World Health Organisation, 2021.
7. Harm, H.C.W., *Health Care's Climate Footprint—How the Health Sector Contributes to the Global Climate Crisis and Opportunities for Action*. 2019, ARUP London, UK.
8. Whitmee, S., et al., *Safeguarding human health in the Anthropocene epoch: report of The Rockefeller Foundation–Lancet Commission on planetary health.* 2015. **386**(10007): p. 1973-2028.
9. Polivka, B.J., R.V. Chaudry, and J. Mac Crawford, *Public Health Nurses' Knowledge and Attitudes Regarding Climate Change.* Environmental Health Perspectives, 2012. **120**(3): p. 321-325.
10. Anåker, A., et al., *Nurses' perceptions of climate and environmental issues: a qualitative study.* Journal of Advanced Nursing (John Wiley & Sons, Inc.), 2015. **71**(8): p. 1883-1891.
11. Xiao, J., et al., *Nurses' knowledge and attitudes regarding potential impacts of climate change on public health in central of China.* International Journal of Nursing Sciences, 2016. **3**(2): p. 158-161.
12. Lilienfeld, E., et al., *Addressing climate change through a nursing lens within the framework of the United Nations Sustainable Development Goals.* Nursing Outlook, 2018. **66**(5): p. 482-494.
13. Aronsson, J., et al., *Awareness and attitudes towards sustainability and climate change amongst students and educators in nursing: A systematic integrative review protocol.* Nursing Open, 2022. **9**(1): p. 839-844.
14. Harden, A.J.F., *Mixed-methods systematic reviews: Integrating quantitative and qualitative findings.* 2010. **25**: p. 1-8.
15. Pearson, A., et al., *A mixed-methods approach to systematic reviews.* 2015. **13**(3): p. 121-131.
16. Sandelowski, M., et al., *Text-in-context: a method for extracting findings in mixed-methods mixed research synthesis studies.* 2013. **69**(6): p. 1428-1437.
17. Stern, C., et al., *Methodological guidance for the conduct of mixed methods systematic reviews.* JBI evidence implementation, 2021. **19**(2): p. 120-129.
18. Page, M.J., et al., *The PRISMA 2020 statement: an updated guideline for reporting systematic reviews.* 2021. **10**(1): p. 1-11.
19. Polit, D. and C. Beck, *Essentials of nursing research: Appraising evidence for nursing practice*. 2020: Lippincott Williams & Wilkins.
20. Moher, D.J.P.r.i.f.s.r. and M.T.P.s. bMJ, *liberati a, Tetzlaff J, altman Dg; The PrisMa group.* 2009. **339**: p. b2535.
21. Munn, Z., et al., *The development of software to support multiple systematic review types: the Joanna Briggs Institute System for the Unified Management, Assessment and Review of Information (JBI SUMARI).* 2019. **17**(1): p. 36-43.
22. Hong, Q.N., et al., *The Mixed Methods Appraisal Tool (MMAT) version 2018 for information professionals and researchers.* 2018. **34**(4): p. 285-291.
23. Voils, C., et al., *A Bayesian method for the synthesis of evidence from qualitative and quantitative reports: the example of antiretroviral medication adherence.* 2009. **14**(4): p. 226-233.
24. Institute, J.B.I.J.A.T.J.B., *The Joanna Briggs Institute Reviewers' manual 2014: methodology for JBI mixed methods systematic reviews.* 2014.
25. Munn, Z., et al., *Evidence transfer: ensuring end users are aware of, have access to, and understand the evidence.* 2018. **16**(2): p. 83-89.

For peer review only

Supplementary material 1. Full search strategies for perception, attitude, awareness and perspective towards sustainability practices in relation to climate change among nurses.

Database: PubMed

#1 "Nurses"[Mesh] OR "Nursing"[Mesh] OR "nurs*"[tw]

#2 "Climate Change"[Mesh] OR "Greenhouse Effect"[Mesh] OR "Carbon Footprint"[Mesh] OR "climate change"[tw] OR "sustainab*"[tw] OR "sustainable healthcare"[tw] OR "environmentally responsible healthcare"[tw] OR "global warming"[tw] OR "environmental responsib*"[tw] OR net zero healthcare [tw]

#3 "Survey*"[tw] OR " questionnaire*"[tw] OR "interview*"[tw] OR "observ*"[tw] OR "case stud*"[tw] OR "focus group*"[tw]

#4 "Social Perception"[Mesh] "Attitude"[Mesh] "Awareness"[Mesh] OR "Opinion*" OR "perce*"[tw] OR "aware*"[tw] OR "perspect*"[tw] OR "attitude*"[tw] OR "belie*"[tw] OR "know*"[tw] OR "pract*"[tw]

#5 ("Qualitative Research"[Mesh] OR "mixed method*"[tw] OR qualitative[tw] OR quantitative[tw]

#1 AND #2 AND #3 AND #4 AND #5

Filters: English

Database: PsycINFO

#1 “nurs*”

#2 “climate change” OR “sustainab*” OR “sustainable healthcare” OR “environmentally responsible healthcare” OR “global warming” OR “environmental responsib*” OR “net zero healthcare”

For peer review only

#3 “Survey*” OR “questionnaire*” OR “interview*” OR “observ*” OR “case stud*” OR “focus group*”

#4 “Opinion*” OR “perce*” OR “aware*” OR “perspect*” OR “attitude*” OR “belie*” OR “sustainable pract*” OR “know*” OR “pract*”

#5 “mixed method*” OR “qualitative” OR “quantitative”

#1 AND #2 AND #3 AND #4 AND #5

Filters: English

Database: CINAHL ULTIMATE

#1 “nurs*”

#2 “climate change” OR “sustainab*” OR “sustainable healthcare” OR “environmentally responsible healthcare” OR “global warming” OR “environmental responsib*” OR “net zero healthcare”

#3 “Survey*” OR “questionnaire*” OR “interview*” OR “observ*” OR “case stud*” OR “focus group*”

#4 “Opinion*” OR “perce*” OR “aware*” OR “perspect*” OR “attitude*” OR “belie*” OR “sustainable pract*” OR “know*” OR “pract*”

#5 “mixed method*” OR “qualitative” OR “quantitative”

#1 AND #2 AND #3 AND #4 AND #5

Filters: English

DATABASE: SCOPUS

For peer review only

#1 nurs*

#2 climate change*, which was seen in the search as climate AND change*

#1 AND #2

Filters: English

The above in SCOPUS produced relevant papers and papers which were also noted during the scoping review/searches.

For peer review only

PRISMA-P (Preferred Reporting Items for Systematic review and Meta-Analysis Protocols) 2015 checklist: recommended items to address in a systematic review protocol*

Section and topic	Item No	Checklist item	Information reported YES	Information reported NO	Line/page Number (s)
**ADMINISTRATIVE INFORMATION**					
Title:					
Identification	1a	Identify the report as a protocol of a systematic review	☒	☐	1
Update	1b	If the protocol is for an update of a previous systematic review, identify as such	☐	☒	
Registration	2	If registered, provide the name of the registry (such as PROSPERO) and registration number	☒	☐	4
Authors:					
Contact	3a	Provide name, institutional affiliation, e-mail address of all protocol authors; provide physical mailing address of corresponding author	☒	☐	1
Contributions	3b	Describe contributions of protocol authors and identify the guarantor of the review	☒	☐	9
Amendments	4	If the protocol represents an amendment of a previously completed or published protocol, identify as such and list changes; otherwise, state plan for documenting important protocol amendments	☐	☒	
Support:					
Sources	5a	Indicate sources of financial or other support for the review	☒	☐	9
Sponsor	5b	Provide name for the review funder and/or sponsor	☒	☐	9
Role of sponsor or funder	5c	Describe roles of funder(s), sponsor(s), and/or institution(s), if any, in developing the protocol	☒	☐	9
**INTRODUCTION**					
Rationale	6	Describe the rationale for the review in the context of what is already known	☒	☐	3
Objectives	7	Provide an explicit statement of the question(s) the review will address with reference to participants, interventions, comparators, and outcomes (PICO)	☒	☐	4
**METHODS**					
Eligibility criteria	8	Specify the study characteristics (such as PICO, study design, setting, time frame) and report characteristics (such as years considered, language, publication status) to be used as criteria for eligibility for the review	☒	☐	4

For peer review only

Information sources	9	Describe all intended information sources (such as electronic databases, contact with study authors, trial registers or other grey literature sources) with planned dates of coverage	☒	☐	5
Search strategy	10	Present draft of search strategy to be used for at least one electronic database, including planned limits, such that it could be repeated	☒	☐	5
Study records:					
Data management	11a	Describe the mechanism(s) that will be used to manage records and data throughout the review	☒	☐	6
Selection process	11b	State the process that will be used for selecting studies (such as two independent reviewers) through each phase of the review (that is, screening, eligibility and inclusion in meta-analysis)	☒	☐	6
Data collection process	11c	Describe planned method of extracting data from reports (such as piloting forms, done independently, in duplicate), any processes for obtaining and confirming data from investigators	☒	☐	7
Data items	12	List and define all variables for which data will be sought (such as PICO items, funding sources), any pre-planned data assumptions and simplifications	☒	☐	4
Outcomes and prioritization	13	List and define all outcomes for which data will be sought, including prioritization of main and additional outcomes, with rationale	☒	☐	7
Risk of bias in individual studies	14	Describe anticipated methods for assessing risk of bias of individual studies, including whether this will be done at the outcome or study level, or both; state how this information will be used in data synthesis	☒	☐	7
Data synthesis	15a	Describe criteria under which study data will be quantitatively synthesised	☐	☒	7
	15b	If data are appropriate for quantitative synthesis, describe planned summary measures, methods of handling data and methods of combining data from studies, including any planned exploration of consistency (such as I^2, Kendall's τ)	☐	☒	7
	15c	Describe any proposed additional analyses (such as sensitivity or subgroup analyses, meta-regression)	☐	☒	7
	15d	If quantitative synthesis is not appropriate, describe the type of summary planned	☒	☐	8
Meta-bias(es)	16	Specify any planned assessment of meta-bias(es) (such as publication bias across studies, selective reporting within studies)	☒	☐	2
Confidence in cumulative evidence	17	Describe how the strength of the body of evidence will be assessed (such as GRADE)	☒	☐	7

*** It is strongly recommended that this checklist be read in conjunction with the PRISMA-P Explanation and Elaboration (cite when available) for important clarification on the items. Amendments to a review protocol should be tracked and dated. The copyright for PRISMA-P (including checklist) is held by the PRISMA-P Group and is distributed under a Creative Commons Attribution Licence 4.0.**

Shamseer L, Moher D, Clarke M, Ghersi D, Liberati A, Petticrew M, Shekelle P, Stewart L, PRISMA-P Group. Preferred reporting items for systematic review and meta-analysis protocols (PRISMA-P) 2015: elaboration and explanation. BMJ. 2015 Jan 2;349(jan02 1):g7647.

For peer review only

For peer review only

BMJ Open

Perceptions, attitudes, awareness and perspectives towards sustainability practices and climate change among nurses: A systematic review protocol

Journal:	*BMJ Open*
Manuscript ID	bmjopen-2023-071965.R2
Article Type:	Protocol
Date Submitted by the Author:	07-Sep-2023

Complete List of Authors:	Akore Yeboah, Ebenezer; Coventry University, Research Centre for Healthcare and Communities<br>Adegboye, Amanda ; Coventry University, Centre for Agroecology, Water and Resilience<br>Kneafsey, Rosie ; Coventry University, Research Centre for Healthcare and Communities

**Primary Subject Heading**:	Nursing
Secondary Subject Heading:	Nursing
Keywords:	PUBLIC HEALTH, Health & safety < HEALTH SERVICES ADMINISTRATION & MANAGEMENT, Systematic Review

SCHOLARONE™
Manuscripts

PERCEPTIONS, ATTITUDES, AWARENESS AND PERSPECTIVES TOWARDS SUSTAINABILITY PRACTICES AND CLIMATE CHANGE AMONG NURSES: A SYSTEMATIC REVIEW PROTOCOL

Ebenezer Akore Yeboah[1] (corresponding author): ebenezer.akore@gmail.com

Amanda Rodrigues Amorim Adegboye[2]: ad6287@coventry.ac.uk

Rosie Kneafsey[1]: aa9398@coventry.ac.uk

1. Research Centre for Healthcare and Communities, Coventry University, UK
2. Centre for Agroecology, Water and Resilience, Coventry University, UK

ABSTRACT

Introduction: Climate change has been described as the most significant threat to humanity and human health to have emerged this century. It is widely accepted that contemporary human activities are the major causes of climate change. It is also acknowledged that damaging human activities could be amenable to change through pro-active environmental behaviours. Healthcare professionals have the potential to promote climate advocacy and mitigation through collective effort and individual actions. However, research suggests that nurses may not be aware of their potential to effect positive action. This review will synthesise evidence regarding nurses' perceptions, attitudes, awareness, and perspectives towards sustainable nursing practices and climate change.

Methods and Analysis: The JBI methodology for mixed methods systematic reviews will be applied to this proposed systematic review. It will be reported in accordance with the Preferred Reporting Items for Systematic Reviews and Meta Analyses (PRISMA) guidelines. CINAHL, PsycINFO, SCOPUS, and PUBMED databases will be searched. Data appraisal will be completed using the JBI and MMAT critical appraisal tool. Data synthesis and integration will follow the JBI convergent integrated approach.

Ethics and Dissemination: In compliance with University ethics requirements for secondary research and post-graduate researchers, ethical approval will be sought from the Coventry University Ethics Committee, UK. Dissemination of findings will be achieved through peer-review publications, conference presentations and seminars with local, national and international audiences.

For peer review only - http://bmjopen.bmj.com/site/about/guidelines.xhtml

Strength and limitations

- This study will be limited to papers published in English.
- There is a risk that relevant information in other languages could be missed.
- Rigour and transparency will be observed in the review process.

For peer review only

INTRODUCTION

Climate change has been described as the most significant threat this century to humanity and the greatest source of harm to human health [1]. Climate change is defined as 'the systematic change in the long term state of the atmosphere over multiple decades or longer'[2]. In the 19th century, natural events such as changes in incoming solar radiation, volcanoes and changes in global biogeochemical cycles were identified by scientists as the major causes of climate change [3]. However, scientific consensus has established that contemporary human activities such as combustion of fossil fuel for energy production and energy use, deforestations, atmospheric aerosols, change in land use and emission of other gases such as water vapour and other greenhouse gases are the major causes of climate change[3].

The impact of climate change on human health is significant and as such is a critical issue [4]. It is widely accepted that the populations which suffer most are those which contribute little to greenhouse gas emissions [5]. Such populations are particularly vulnerable due to geographic placement, social isolation, and economic disadvantage. The documented impact of heatwaves, floods, drought and climate change related disease have been devastating. For example, it is estimated that climate change will cause an additional 250,000 deaths across the globe between 2030 and 2050, from heat exhaustion in elderly people, malnutrition among children, malaria and diarrhoea [4].

Globally, health professionals are acknowledging and responding to the damage caused by climate change and the impact on health [6]. At the same time, healthcare systems themselves account for 4.4% of the global net CO2 emissions thus rendering significant climate impact, both through carbon emissions and use of carbon compounds for human health benefit. Indeed,

For peer review only - http://bmjopen.bmj.com/site/about/guidelines.xhtml

it has been identified that if the healthcare sector were a country, it would rank the fifth-largest producer of greenhouse gases on the planet [7]. More specifically for the UK, the National Health Service (NHS) accounts for 5.4% of the UK greenhouse emissions and this is higher than the global average of the healthcare system [7].

Healthcare professionals have the potential to promote climate advocacy and mitigation through collective effort and individual actions[8]. However, research suggests that nurses may not be aware of the potential to effect positive action [9]. A study in Sweden identified that whilst nurses exhibited an understanding of environmental issues in their local setting, a comprehensive global perspective on climate change and environmental issues was less developed [10]. In contrast, a study in China showed that whilst 76% of nurses knew that climate change would affect public health, more than 50% did not know that their own local nursing practice could be related to carbon compound emissions and subsequently climate change [11]. Across continents, a study including American nurses found that they clearly believed the present threat of climate change and recognised their responsibility as health workers to address the health effects of climate change [9].

It is known that nurses form the largest proportion of the healthcare workforce and are a significant professional force. As such, practicing environmentally responsible healthcare as well as embedding the concepts of sustainability and climate actions into nursing curricula could be important in the fight against climate change [12]. There exists an ongoing integrative review protocol exploring awareness and attitudes of student nurses and educators toward sustainability and climate change [13]. However, there is no systematic review focusing on the perspectives of registered/ qualified nurses. Studies have used different samples, research designs and have reached different conclusions. Therefore, this review aims to systematically synthesise evidence concerning nurses' perceptions, attitudes, awareness, and perspectives of sustainable nursing practice and climate change.

METHOD

For peer review only - http://bmjopen.bmj.com/site/about/guidelines.xhtml

DESIGN

A mixed method systematic review will be conducted to answer the research question related to the phenomena of interest in this study. Mixed method systematic review merges and homogenise findings of quantitative and qualitative studies within a single review [14]. Mixed methods reviews are useful in producing systematic reviews of direct importance or significance to policy makers and practitioners [15]. This design is also appropriate and suitable when the researcher wants to broaden the conceptualisation of evidence that is methodologically inclusive. It synthesizes evidence that will be available to a wider range of consumers. Hence, this approach enables a comprehensiveness exploration of the literature related to nursing, climate change and sustainability [16]. The updated JBI methodology for mixed methods systematic reviews [17] will be used for this systematic review. It will be reported in accordance with the Preferred Reporting Items for Systematic Reviews and Meta Analyses (PRISMA) guidelines [18]. The review protocol is registered in the Open Science Framework (OSF) and registration DOI 10.17605/OSF.IO/8H3TC.

Identification of problem

The SPIDER (Sample, Phenomenon of Interest, Design, Evaluation and Research type) framework has been used to formulate the research question and determine the criteria for inclusion and exclusion. Table 1.

Table 1. Inclusion and exclusion criteria in the literature search

Domains	Inclusion	Exclusion
Sample	▪ Studies/ publication /articles focused on registered or qualified nurses regardless of work setting<br>▪ Can include other health professionals as long as findings reported nurses separately.	▪ Studies/ publication /articles on climate change focusing on nursing students and/or nursing educators.<br>▪ Studies on other healthcare professional e.g. care assistant, doctor etc.
Phenomenon of interest	▪ Climate change, environmental sustainability, net zero healthcare and nursing practice or any related terms to these	▪ Studies that do not include climate change or sustainable environmental practice or any related term

For peer review only - http://bmjopen.bmj.com/site/about/guidelines.xhtml

Design	▪ Empirical research studies using any research design e.g., interview, survey, focus group etc.<br>▪ Published literature, governmental reports, technical documents, conference abstracts, manuals, on net zero healthcare (or climate change) with relation to nursing (which are primary or empirical study).	▪ Commentaries, press releases, speeches, letters to the editor are excluded<br>▪ All publications relating to the phenomena of interest which are not empirical research.
Evaluation	▪ Studies exploring the perception, OR attitude, OR awareness (or knowledge) OR the perspective on climate change and related terms	
Research type	❖ Qualitative, quantitative or mixed methods approaches to primary research	❖ Secondary research

For peer review only

All studies regardless of the date of publication will be included. Studies across the globe or any country will be included but limited to publications in English.

Searching Literature

Search strategy

A preliminary scoping search was done to identify articles on the study topic. A full search strategy was developed after filtering through various titles and abstract of relevant articles and their index terms. Table 2, (see also supplementary file).

Information source

The databases to be searched include; CINAHL, PsycINFO, SCOPUS and PUBMED. These data bases have been selected due to their broad range of literature in the fields of health and social sciences, nursing and climate change.

Table 2: Search terms

	Concept	Truncated terms
Sample	"nursing" OR "nurses"	"nurs*"

For peer review only - http://bmjopen.bmj.com/site/about/guidelines.xhtml

Phenomenon of interest	"climate change" OR "sustainability" OR "sustainable healthcare" OR "environmentally responsible healthcare" OR "global warming" OR "environmental responsibility" OR "net zero healthcare"	"climate change" OR "sustainab*" OR "sustainable healthcare" OR "environmentally responsible healthcare" OR "global warming" OR "environmental responsib*" OR "net zero healthcare"
Design	"Survey" OR " questionnaire" OR "interviews" OR "observational study" OR "case study" OR "focus group discussion"	"Survey*" OR " questionnaire*" OR "interview*" OR "observ*" OR "case stud*" OR "focus group*"
Evaluation	"perception" OR "awareness" OR "perspectives" OR "attitude" OR "beliefs" OR "knowledge" OR "opinion" OR "sustainable practice" OR "practice"	"Opinion*" OR "perce*" OR "aware*" OR "perspect*" OR "attitude*" OR "belie*" OR "sustainable pract*" OR "know*" OR "pract*"
Research type	"mixed methods" OR "qualitative" OR "quantitative"	"mixed method*" OR "qualitative" OR "quantitative"

For peer review only

Study selection

The search results from each database will be imported in EndNote reference manager to remove duplicates and then transferred to Rayyan web tool where screening based on the inclusion and exclusion criteria will take place. In the initial phase, 20% of the studies will be screened by the three reviewers (EAY, AA, RK) independently based on title and abstract. This independent screening process will reduce the risk of bias and ensure consistency in the review process [19]. Once the initial screening phase is done, two reviewers (EAY, AA) will continue the title and abstract screening on the remaining studies. Any studies selected at this stage will then be subjected to full text screening by the same two reviewers (EAY, AA) [13]. Any differences between the two reviewers will be resolved through discussion or by the third reviewer (RK).

For peer review only - http://bmjopen.bmj.com/site/about/guidelines.xhtml

During this second search, bibliography or reference lists of the studies which were included will be manually reviewed. Any reason for excluding a paper during the full text assessment that does not meet the inclusion criteria will be recorded and reported in the systematic review findings. In instances of disagreement between the two reviewers in the second phase of screening the full text, consensus will be reached through discussion or third reviewer. All members of the review team (EAY, AA, RK) will inspect and assess the final list of papers that met the inclusion criteria before the progressing to the next stages of the review methodology. A PRISMA flow diagram will be designed to show the completed search [20].

Assessment of methodological quality.

The standardised JBI qualitative critical appraisal tool will be used to assess qualitative papers that meet the inclusion criteria [21]. The quantitative and mixed method papers will be methodologically appraised with the Mixed Methods Assessment Tool (MMAT) version 2018 [22]. These methodological appraisals will be done by the two review members (EAY, AA) independently, prior to data extraction.

The quality appraisal of the included papers will be compared and discussed to determine the overall quality score and in situations of dissimilarity, it would be discussed collaboratively among the review team, employing triangulation or an independent reviewer as a measure of credibility or trustworthiness [19]. This will be done to reduce any risks of bias and provide compelling evidence for the study. Regardless of the methodological quality, all included studies will undergo data extraction and synthesis (where possible). The results of the appraisal will summarised in a table and reported in the review.

Data extraction

Both the qualitative and quantitative data will be extracted from the included papers by two reviewers (EAY, AA) using the standardised JBI data extraction tools [21]. Any discrepancies will be resolved through discussion among the two reviewers or with triangulation. Authors of papers will be contacted for missing or additional data, if required [21].

Data transformation

For the purpose of integration, the extracted data will need to be transformed into mutually compatible forms [23]. In this review, the retrieved quantitative data will undergo qualitizing

For peer review only - http://bmjopen.bmj.com/site/about/guidelines.xhtml

[24]. In this way, extracted quantitative data will be converted into qualitative data. This will involve conversion of quantitative data into textual description thus themes, categories etc, in order to be able to answer the review question. Qualitizing is chosen to reduce the risk of error as opposed to quantitizing [24].

Data synthesis and integration

The JBI convergent integrated approach will be adopted to synthesize the transformed data [21]. This involves combining the extracted data from quantitative and qualitative or mixed method studies. The qualitized data will be assembled with qualitative information and then categorised. The categorised data will be pooled together based on similarity in meaning to produce a set of integrated finding [17].

Patient and Public Involvement

None

Discussion

This mixed method review will enrich the conceptualisation of evidence so that methodologically inclusive and synthesized data will be available to the nursing community and policy makers. This review will synthesize the existing body of literature related to nurses' perceptions, attitudes, awareness and perspectives towards sustainability practices in relation to climate change. The findings could be significant to hospital managers, nurse leaders and green or sustainability champions when planning environmentally responsible healthcare projects. For example, relating to sustainable healthcare practices, meeting net-zero goals or greening hospitals. Since nurses form the largest part of the healthcare workforce and present a significant professional force, translating evidence from research into practice and promoting nurse engagement in this aspect of healthcare could be impactful [12, 25]. The review will be disseminated in journals, conferences, and with relevant institutions to improve practice and share knowledge for the benefit of the global population. Climate change and sustainability are becoming ubiquitous in all spheres of life as the world experiences the impact of this phenomenon.

Conclusion

For peer review only - http://bmjopen.bmj.com/site/about/guidelines.xhtml

The findings of this mixed method review will be to raise awareness of sustainable healthcare actions and identify opportunities to accelerate the implementation of environmentally responsible nursing practices. The review will also promote dialogue with policy makers, nursing and healthcare leaders regarding the benefit of further mobilising nurse engagement in climate actions and policies.

Ethics

Ethical approval will be sought from the University Ethics Committee, Coventry University, UK.

Acknowledgement

Thank you to all the staff of Research Centre for Healthcare and Communities, Coventry University, for supporting the PhD study.

Funding

Thank you to the Research Centre for Healthcare and Communities, Coventry University, for funding the PhD study. No fund/grant number.

Conflict of interest

The authors declared no potential conflicts of interest.

Author Contributions

Ebenezer Akore Yeboah, Amanda Rodrigues Amorim Adegboye, and Rosie Kneafsey, all contributed to the conception, design, drafting manuscript, revising and approval of the final version to be published. However, Ebenezer Akore Yeboah is accountable for all aspect of the work.

Reference

1. Watts, N., et al., *The 2020 report of the Lancet Countdown on health and climate change: responding to converging crises.* 2021. **397**(10269): p. 129-170.
2. Knowlton, K., C. Sorensen, and J. Lemery, *Global climate change and human health: from science to practice*. 2021: John Wiley & Sons.
3. Geneva, S.J.W.G.I.C.t.t.I.F.A.R.C.C., *Intergovernmental Panel on Climate Change, 2014.* 2013. **8**.

For peer review only - http://bmjopen.bmj.com/site/about/guidelines.xhtml

4. WHO, *climate change.* 2022.
5. Lemery, J., et al., *The great procrastination*. 2014, HeinOnline. p. 1.
6. WHO, *climate change and health.* World Health Organisation, 2021.
7. Harm, H.C.W., *Health Care's Climate Footprint—How the Health Sector Contributes to the Global Climate Crisis and Opportunities for Action*. 2019, ARUP London, UK.
8. Whitmee, S., et al., *Safeguarding human health in the Anthropocene epoch: report of The Rockefeller Foundation–Lancet Commission on planetary health.* 2015. **386**(10007): p. 1973-2028.
9. Polivka, B.J., R.V. Chaudry, and J. Mac Crawford, *Public Health Nurses' Knowledge and Attitudes Regarding Climate Change.* Environmental Health Perspectives, 2012. **120**(3): p. 321-325.
10. Anåker, A., et al., *Nurses' perceptions of climate and environmental issues: a qualitative study.* Journal of Advanced Nursing (John Wiley & Sons, Inc.), 2015. **71**(8): p. 1883-1891.
11. Xiao, J., et al., *Nurses' knowledge and attitudes regarding potential impacts of climate change on public health in central of China.* International Journal of Nursing Sciences, 2016. **3**(2): p. 158-161.
12. Lilienfeld, E., et al., *Addressing climate change through a nursing lens within the framework of the United Nations Sustainable Development Goals.* Nursing Outlook, 2018. **66**(5): p. 482-494.
13. Aronsson, J., et al., *Awareness and attitudes towards sustainability and climate change amongst students and educators in nursing: A systematic integrative review protocol.* Nursing Open, 2022. **9**(1): p. 839-844.
14. Harden, A.J.F., *Mixed-methods systematic reviews: Integrating quantitative and qualitative findings.* 2010. **25**: p. 1-8.
15. Pearson, A., et al., *A mixed-methods approach to systematic reviews.* 2015. **13**(3): p. 121-131.
16. Sandelowski, M., et al., *Text-in-context: a method for extracting findings in mixed-methods mixed research synthesis studies.* 2013. **69**(6): p. 1428-1437.
17. Stern, C., et al., *Methodological guidance for the conduct of mixed methods systematic reviews.* JBI evidence implementation, 2021. **19**(2): p. 120-129.
18. Page, M.J., et al., *The PRISMA 2020 statement: an updated guideline for reporting systematic reviews.* 2021. **10**(1): p. 1-11.
19. Polit, D. and C. Beck, *Essentials of nursing research: Appraising evidence for nursing practice*. 2020: Lippincott Williams & Wilkins.
20. Moher, D.J.P.r.i.f.s.r. and M.T.P.s. bMJ, *liberati a, Tetzlaff J, altman Dg; The PrisMa group.* 2009. **339**: p. b2535.
21. Munn, Z., et al., *The development of software to support multiple systematic review types: the Joanna Briggs Institute System for the Unified Management, Assessment and Review of Information (JBI SUMARI).* 2019. **17**(1): p. 36-43.
22. Hong, Q.N., et al., *The Mixed Methods Appraisal Tool (MMAT) version 2018 for information professionals and researchers.* 2018. **34**(4): p. 285-291.
23. Voils, C., et al., *A Bayesian method for the synthesis of evidence from qualitative and quantitative reports: the example of antiretroviral medication adherence.* 2009. **14**(4): p. 226-233.
24. Institute, J.B.I.J.A.T.J.B., *The Joanna Briggs Institute Reviewers' manual 2014: methodology for JBI mixed methods systematic reviews.* 2014.
25. Munn, Z., et al., *Evidence transfer: ensuring end users are aware of, have access to, and understand the evidence.* 2018. **16**(2): p. 83-89.

For peer review only

Supplementary material 1. Full search strategies for perception, attitude, awareness and perspective towards sustainability practices in relation to climate change among nurses.

Database: PubMed

#1 "Nurses"[Mesh] OR "Nursing"[Mesh] OR "nurs*"[tw]

#2 "Climate Change"[Mesh] OR "Greenhouse Effect"[Mesh] OR "Carbon Footprint"[Mesh] OR "climate change"[tw] OR "sustainab*"[tw] OR "sustainable healthcare"[tw] OR "environmentally responsible healthcare"[tw] OR "global warming"[tw] OR "environmental responsib*"[tw] OR net zero healthcare [tw]

#3 "Survey*"[tw] OR " questionnaire*"[tw] OR "interview*"[tw] OR "observ*"[tw] OR "case stud*"[tw] OR "focus group*"[tw]

#4 "Social Perception"[Mesh] "Attitude"[Mesh] "Awareness"[Mesh] OR "Opinion*" OR "perce*"[tw] OR "aware*"[tw] OR "perspect*"[tw] OR "attitude*"[tw] OR "belie*"[tw] OR "know*"[tw] OR "pract*"[tw]

#5 ("Qualitative Research"[Mesh] OR "mixed method*"[tw] OR qualitative[tw] OR quantitative[tw]

#1 AND #2 AND #3 AND #4 AND #5

Filters: English

Database: PsycINFO

#1 “nurs*”

#2 “climate change” OR “sustainab*” OR “sustainable healthcare” OR “environmentally responsible healthcare” OR “global warming” OR “environmental responsib*” OR “net zero healthcare”

For peer review only

#3 “Survey*” OR “questionnaire*” OR “interview*” OR “observ*” OR “case stud*” OR “focus group*”

#4 “Opinion*” OR “perce*” OR “aware*” OR “perspect*” OR “attitude*” OR “belie*” OR “sustainable pract*” OR “know*” OR “pract*”

#5 “mixed method*” OR “qualitative” OR “quantitative”

#1 AND #2 AND #3 AND #4 AND #5

Filters: English

Database: CINAHL ULTIMATE

#1 “nurs*”

#2 “climate change” OR “sustainab*” OR “sustainable healthcare” OR “environmentally responsible healthcare” OR “global warming” OR “environmental responsib*” OR “net zero healthcare”

#3 “Survey*” OR “questionnaire*” OR “interview*” OR “observ*” OR “case stud*” OR “focus group*”

#4 “Opinion*” OR “perce*” OR “aware*” OR “perspect*” OR “attitude*” OR “belie*” OR “sustainable pract*” OR “know*” OR “pract*”

#5 “mixed method*” OR “qualitative” OR “quantitative”

#1 AND #2 AND #3 AND #4 AND #5

Filters: English

For peer review only

DATABASE: SCOPUS

#1 nurs*

#2 climate change*, which was seen in the search as climate AND change*

#1 AND #2

Filters: English

The above in SCOPUS produced relevant papers and papers which were also noted during the scoping review/searches.

For peer review only

PRISMA-P (Preferred Reporting Items for Systematic review and Meta-Analysis Protocols) 2015 checklist: recommended items to address in a systematic review protocol*

Section and topic	Item No	Checklist item	Information reported YES	Information reported NO	Line/page Number (s)
**ADMINISTRATIVE INFORMATION**					
Title:					
Identification	1a	Identify the report as a protocol of a systematic review	☒	☐	1
Update	1b	If the protocol is for an update of a previous systematic review, identify as such	☐	☒	
Registration	2	If registered, provide the name of the registry (such as PROSPERO) and registration number	☒	☐	4
Authors:					
Contact	3a	Provide name, institutional affiliation, e-mail address of all protocol authors; provide physical mailing address of corresponding author	☒	☐	1
Contributions	3b	Describe contributions of protocol authors and identify the guarantor of the review	☒	☐	9
Amendments	4	If the protocol represents an amendment of a previously completed or published protocol, identify as such and list changes; otherwise, state plan for documenting important protocol amendments	☐	☒	
Support:					
Sources	5a	Indicate sources of financial or other support for the review	☒	☐	9
Sponsor	5b	Provide name for the review funder and/or sponsor	☒	☐	9
Role of sponsor or funder	5c	Describe roles of funder(s), sponsor(s), and/or institution(s), if any, in developing the protocol	☒	☐	9
**INTRODUCTION**					
Rationale	6	Describe the rationale for the review in the context of what is already known	☒	☐	3
Objectives	7	Provide an explicit statement of the question(s) the review will address with reference to participants, interventions, comparators, and outcomes (PICO)	☒	☐	4
**METHODS**					
Eligibility criteria	8	Specify the study characteristics (such as PICO, study design, setting, time frame) and report characteristics (such as years considered, language, publication status) to be used as criteria for eligibility for the review	☒	☐	4

For peer review only

Information sources	9	Describe all intended information sources (such as electronic databases, contact with study authors, trial registers or other grey literature sources) with planned dates of coverage	☒	☐	5
Search strategy	10	Present draft of search strategy to be used for at least one electronic database, including planned limits, such that it could be repeated	☒	☐	5
Study records:					
Data management	11a	Describe the mechanism(s) that will be used to manage records and data throughout the review	☒	☐	6
Selection process	11b	State the process that will be used for selecting studies (such as two independent reviewers) through each phase of the review (that is, screening, eligibility and inclusion in meta-analysis)	☒	☐	6
Data collection process	11c	Describe planned method of extracting data from reports (such as piloting forms, done independently, in duplicate), any processes for obtaining and confirming data from investigators	☒	☐	7
Data items	12	List and define all variables for which data will be sought (such as PICO items, funding sources), any pre-planned data assumptions and simplifications	☒	☐	4
Outcomes and prioritization	13	List and define all outcomes for which data will be sought, including prioritization of main and additional outcomes, with rationale	☒	☐	7
Risk of bias in individual studies	14	Describe anticipated methods for assessing risk of bias of individual studies, including whether this will be done at the outcome or study level, or both; state how this information will be used in data synthesis	☒	☐	7
Data synthesis	15a	Describe criteria under which study data will be quantitatively synthesised	☐	☒	7
	15b	If data are appropriate for quantitative synthesis, describe planned summary measures, methods of handling data and methods of combining data from studies, including any planned exploration of consistency (such as I^2, Kendall's τ)	☐	☒	7
	15c	Describe any proposed additional analyses (such as sensitivity or subgroup analyses, meta-regression)	☐	☒	7
	15d	If quantitative synthesis is not appropriate, describe the type of summary planned	☒	☐	8
Meta-bias(es)	16	Specify any planned assessment of meta-bias(es) (such as publication bias across studies, selective reporting within studies)	☒	☐	2
Confidence in cumulative evidence	17	Describe how the strength of the body of evidence will be assessed (such as GRADE)	☒	☐	7

*** It is strongly recommended that this checklist be read in conjunction with the PRISMA-P Explanation and Elaboration (cite when available) for important clarification on the items. Amendments to a review protocol should be tracked and dated. The copyright for PRISMA-P (including checklist) is held by the PRISMA-P Group and is distributed under a Creative Commons Attribution Licence 4.0.**

Shamseer L, Moher D, Clarke M, Ghersi D, Liberati A, Petticrew M, Shekelle P, Stewart L, PRISMA-P Group. Preferred reporting items for systematic review and meta-analysis protocols (PRISMA-P) 2015: elaboration and explanation. BMJ. 2015 Jan 2;349(jan02 1):g7647.

For peer review only

For peer review only
